# Feasibility, Safety and Efficacy of a Virtual Reality Exergame System to Supplement Upper Extremity Rehabilitation Post-Stroke: A Pilot Randomized Clinical Trial and Proof of Principle

**DOI:** 10.3390/ijerph17010113

**Published:** 2019-12-23

**Authors:** Nahid Norouzi-Gheidari, Alejandro Hernandez, Philippe S. Archambault, Johanne Higgins, Lise Poissant, Dahlia Kairy

**Affiliations:** 1 School of Physical and Occupational Therapy, McGill University, Montreal, QC H3G 1Y5, Canada; philippe.archambault@mcgill.ca; 2 Feil/Oberfeld/CRIR Research Centre, Jewish Rehabilitation Hospital site of CISSS-Laval, Laval, QC H7V 1R2, Canada; 3 CRIR Research Centre, IURDPM site of CIUSSS-Montreal, Montreal, QC H3S 2J4, Canada; alejandro.hernandez@mail.mcgill.ca (A.H.); johanne.higgins@umontreal.ca (J.H.); lise.poissant@umontreal.ca (L.P.); dahlia.kairy@umontreal.ca (D.K.); 4 School of Rehabilitation, Université de Montréal, Montreal, QC H3N 1X7, Canada

**Keywords:** exergames, virtual reality, Kinect, stroke, rehabilitation, upper extremity, video games

## Abstract

(1) Background: Increasing the amount of therapy time has been shown to improve motor function in stroke survivors. However, it is often not possible to increase the amount of therapy time provided in the current one-on-one therapy models. Rehabilitation-based virtual reality exergame systems, such as Jintronix, can be offered to stroke survivors as an adjunct to traditional therapy. The goal of this study was to examine the safety and feasibility of providing additional therapy using an exergame system and assess its preliminary clinical efficacy. (2) Methods: Stroke survivors receiving outpatient rehabilitation services participated in this pilot randomized control trial in which the intervention group received 4 weeks of exergaming sessions in addition to traditional therapy sessions. (3) Results: Nine subjects in the intervention and nine subjects in the control group completed the study. The intervention group had at least two extra sessions per week, with an average duration of 44 min per session and no serious adverse events (falls, dizziness, or pain). The efficacy measures showed statistically meaningful improvements in the activities of daily living measures (i.e., MAL-QOM (motor activity log-quality of movement) and both mobility and physical domains of the SIS (stroke impact scale) with mean difference of 1.0%, 5.5%, and 6.7% between the intervention and control group, respectively) at post-intervention. (4) Conclusion: Using virtual reality exergaming technology as an adjunct to traditional therapy is feasible and safe in post-stroke rehabilitation and may be beneficial to upper extremity functional recovery.

## 1. Introduction

Hemiplegia, weakness of one side of the body, is a common consequence of stroke that can lead to significant functional impairments, including loss of arm function that occurs in up to 85% of stroke survivors [1]. The impact of arm-related limitations on activities of daily living, leisure activities, or work is significant as the arm plays a central role in a person’s life from the ability to perform basic activities of daily life to carrying out family and social roles. Guidelines indicate that rehabilitation can improve upper extremity (UE) motor control and functional status post-stroke [2]. Intense and repetitive training post-stroke is widely recognized as being essential to maximize recovery and promote plasticity in the central nervous system [2,3,4,5,6]. However, in reality, the feasibility of providing intensive rehabilitation may be limited in a rehabilitation setting by a lack of resources or motivation from the patient [7]. Furthermore, stroke survivors discharged from acute care receive physical and occupational therapy services primarily in rehabilitation centers, on an in- and out-patient basis or at home, but budgetary constraints and shortage of specialized health care professionals limit accessibility to much needed rehabilitation services.

New models of service delivery and maximizing the use of existing resources are therefore essential. With the emergence of interactive technologies or “gamified” products, innovative treatment strategies are being developed. Virtual reality (VR) and computer games are recent technologies that, as they become more accessible and affordable [8], are increasingly being used in rehabilitation to allow patients to engage in repetitive practice of specific tasks. The so-called exergames or exergaming reflect the idea of exercising through computer games. A number of published reviews and meta-analyses have examined the use of VR and video games for post-stroke rehabilitation, focusing on or including UE rehabilitation [3,4,6,8,9,10,11].

Saposnik and his colleagues [9] examined the effectiveness of rehabilitation using VR on UE motor function post-stroke in a meta-analysis study. In total, 11 of the 12 included studies (5 randomized controlled trials and 7 pre-post interventions) showed a benefit on the primary outcome, with the pooled randomized controlled trials showing a 4.9 higher chance of improvement in motor impairment when VR was used in comparison to control. Similarly, another meta-analysis study included a subgroup of studies related to UE function [6] and showed that VR had a significant effect on arm function (seven studies) and activities of daily living (ADL) (three studies) in comparison to control groups. The updated Cochrane Review of the meta-analysis concluded that virtual reality has a statistically significant effect on upper limb function (based on 12 studies) and ADL outcome (based on 8 studies) [12]. Another meta-analysis looked at the effect of virtual environments and commercial games on each category of the International Classification of Function, Disability, and Health (ICF), i.e., body function, activity, and participation, in post-stroke rehabilitation [11]. The authors reported that there was a significant effect of using virtual environments in improving all three ICF categories and that these outcome improvements by VR rehabilitation are moderately higher than conventional therapy [11]. However, there is still insufficient evidence regarding the best dose of therapy, timing, and types of programs. In addition, few studies report the users’ perception of these technologies, for both patients and therapists, although Lange and her colleagues [4] discussed the importance of activity enjoyment in their review. Celinder and Peoples [13] studied patients’ experiences through interviews and observation of patients using the Wii Sports for in-patient rehabilitation post-stroke. Most patients reported that the Wii provides them with variety, more stimulation, and more meaningful activities in their rehabilitation. Overall, studies agree that there is limited but promising findings that VR and video games, when combined with conventional rehabilitation, have a positive impact on recovery post-stroke. Furthermore, the literature in the field of technology adoption suggests that the user’s perception plays an important role in the actual use of the technology [14].

The Jintronix system (Jintronix Inc., www.jintronix.com), based on the Microsoft Kinect technology, is one such product that allows patients who have had a stroke to train UE movements independently using challenging and engaging programs tailored to their level of ability. This exergame system allows therapists to objectively track patients’ activities during their recovery process and to adjust, as required, the training program. Graded tasks that challenge and provide feedback to the patient can optimize motor learning [2,15]; this system has embedded this concept in their exergames. In addition, eventually, the tracking functionality that the exergame system offers could allow patients to train without direct therapist supervision, thereby offering interesting opportunities for increased dosage of in-clinic rehabilitation as well as for home-based tele-rehabilitation. In other words, following a stroke, people could participate in a more intensive rehabilitation training without increasing staffing. That being said, prior to using this exergame system in a clinical setting when used as an adjunct to conventional therapy, its feasibility, safety, and efficacy must be assessed.

The goals of this pilot study were to assess the feasibility and safety of the rehabilitation exergaming system and to provide preliminary evidence regarding its clinical efficacy for UE functional recovery post-stroke as a supplement to conventional rehabilitation services, as proposed by Saposnik and colleagues [9]. The results of this study will serve as a basis for a larger multicenter trial, in order to determine the effectiveness of exergame systems as a supplement to UE therapy post-stroke.

## 2. Methods

### 2.1. Study Design

A single-blind (evaluator was blind to group assignment) two-arm pilot randomized clinical trial [16] was used in this study with a ‘pre-post-follow up’ (PPF) design. The control group only received usual rehabilitation services while the intervention group had both usual rehabilitation services and additional training with the rehabilitation exergaming system.

### 2.2. Participants and Settings

Participants were chosen among stroke survivors, who were receiving out-patient rehabilitation services but remained with UE motor deficits, and were randomly divided into two groups (using a sealed envelope by the recruiter): An intervention group and a control group. The inclusion criteria were (1) having had an ischemic or hemorrhagic stroke for the first time; (2) having residual mild to moderate UE impairment (score 3–6 on the Chedoke–McMaster arm component [17]); (3) being in subacute or chronic stage; and (4) receiving usual out-patient rehabilitation services at one of the two selected rehabilitation sites, i.e., Institut de réadaptation Gingras-Lindsay-de-Montréal and Jewish Rehabilitation Hospital, all located in the greater Montreal area in Canada. The exclusion criteria consisted of (1) having severe cognitive or communication deficits; (2) having visual impairments; (3) having any medical contraindication for shoulder movements; (4) having severe balance deficits limiting sitting safely independently; (5) having previous UE impairment limiting potential recovery; and (6) having any other impairment that limited use of the VR system. All subjects gave their informed consent for inclusion before they participated in the study. The study was conducted in accordance with the Declaration of Helsinki, and the protocol was approved by the Research Ethics Board of Centre for Interdisciplinary Research in Rehabilitation of Greater Montreal (CRIR-795-0113).

### 2.3. The Rehabilitation Exergaming System

**Figure 1:**
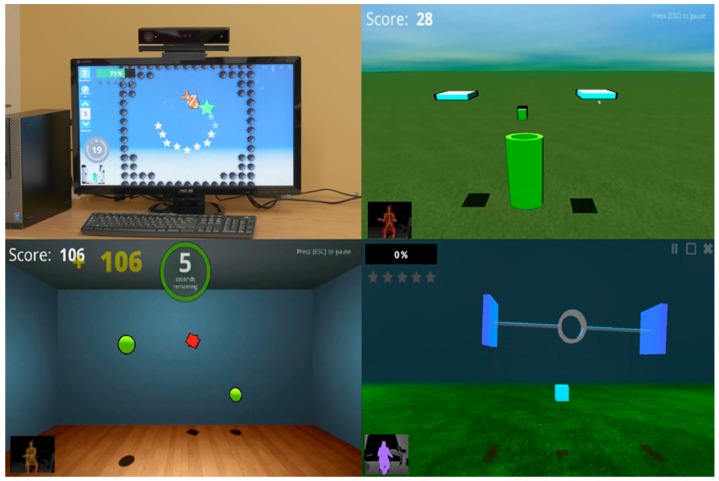
The Jintronix rehabilitation exergaming system.

The Jintronix system was used as the rehabilitation exergaming system in this study. The company has collaborated closely with rehabilitation researchers, clinicians, and patients to develop this system as a unique, engaging, and effective software solution, providing an affordable means of receiving physical and cognitive rehabilitation. This system is an interactive exergame based on the Kinect camera [18], a marker-less motion tracking system (Figure 1). It differs from systems, such as the Nintendo Wii, which requires a wireless controller to detect the person’s movement [19]. Without wearing, holding, or using any sensor, the rehabilitation exergaming system uses the Kinect camera to track the upper and lower body movements in real time; this includes the person’s head, trunk, arm, shoulder, elbow, wrist, and lower body movements on both sides when sitting or standing. The tracked motions are displayed in the gaming environment tailored for therapy. The system provides repeated unilateral and bilateral UE training in all planes, at customizable difficulty levels: Speed, target size, precision, and predictability are all elements that can be programmed by the therapist based on the patient’s abilities. It offers a choice of five UE activities performed against gravity: (1) Tracing a horizontally or vertically oriented path; (2) reaching for a target; (3) moving the hands together to catch, carry, and drop objects; (4) clapping both hands to catch an object between the two hands; and (5) selecting and moving kitchen objects. The exergames do not require or take into account finger movements. Hence, virtual objects are grasped and released when the hand approaches them or moves away from them, and participants are not required to open or close their hands. Patients’ performance on each activity is recorded, allowing review by therapists at a later time to adjust the training program using a clinician portal website. For this study, all activities were done while sitting. At two of the rehabilitation centers, the rehabilitation exergaming system was set up using a desktop computer and a Kinect camera. Previous pilot studies by members of the team have shown that the system can reliably track arm movements and have provided data on clinicians’ and users’ perception of its relevance and potential usefulness for rehabilitation [20].

### 2.4. Study Procedure

All study participants were receiving occupational and/or physical therapy services as prescribed to them post-stroke in the out-patient program at one of the two rehabilitation sites. Services were provided by a multidisciplinary team according to the patient’s needs, with occupational and physical therapy services provided two to three times a week. Those randomly assigned to the intervention group received extra sessions of exergaming to train unilateral and bilateral arm movements in addition to conventional therapy. Each stroke subject in the intervention group used the exergame system two to three times per week, 30 min per session (excluding preparation and other interactions with the system), for four weeks. Rest between therapy and gaming sessions was ensured. At both centers, a therapist (same for all participants in that center), trained on how to use the rehabilitation exergaming system, was always present during the exergaming session. The role of therapists was only limited to adjusting the settings of the system and monitoring the patients’ progress. The intensity and choice of the exergame activities were determined by the therapists based on the patient’s abilities, interests, motivation, and fatigue. At the start of the session, subjects were seated on the chair in front of the system, which was then calibrated based on the subject’s arm range of motion by moving their arms in the sagittal and horizontal planes. This would determine the reaching space of the virtual environment and target placement for the activities. The therapist chose the difficulty levels of the exergames and made sure that the subject played all 5 exergames at least once in each session. During each exergame, comments would pop up based on subject’s performance on the screen. After each trial, a score was presented to the subject according to their performance level and stored into a database. The therapists were instructed to let patients move to a higher level in each exergame if a minimum score of 70% was achieved; however, the decision was solely made by the therapists who considered patients’ performance and motivation as well. During the session, fatigue and pain were monitored using the Borg scale (0–10) and a VAS (0–10), respectively. If there was any report of increased level of pain or fatigue, subjects were given rest periods. At the end of each session, the therapist could view an overall performance report and track the subject’s progress. Within one week after the last session, the final evaluation was conducted. Follow-up measurements were done 4 weeks after the last session. All evaluations were done by two blind evaluators (one at each center) who were trained on the administration of the measures.

### 2.5. Outcome Measures

In both groups, baseline characteristics were collected, which included age, gender, handedness, stroke characteristics (onset, type, side), level of disability based on the Chedoke–McMaster Upper Extremity [17], and comorbid conditions. Three sets of outcome measures were recorded: Feasibility, safety, and efficacy measures. The feasibility measures were related to the performance of the stroke subjects in the rehabilitation exergaming system and consisted of the number and duration of sessions, the time spent by the therapist assisting the patient, the time spent on each exergame, the actual activity, and movements performed as compared to the intended movement. The safety indicators were any occurrence of adverse events, such as increased pain, falls, motion sickness, dizziness, exertion, fatigue, and headaches, when patients engaged with the system.

The outcome measures used for clinical efficacy were: (1) UE motor function (measured by the Fugl–Meyer Assessment-UE (FMA-UE) [21] and the Box and Block test (BBT) [22]); (2) self-reported health status (measured by Stroke Impact Scale (SIS) [23]); and (3) self-reported measure of UE use (measured by Motor Activity Log (MAL) [24]). These were measured at three time points: Pre- (baseline) or T0, post-intervention or T1, and 4-week follow-up or T2 for both groups. The assessors were blind to patient randomization and were not involved in the interventions. The FMA is a performance-based motor impairment index that measures motor recovery post-stroke. The BBT is a quick and simple test that measures unilateral gross manual dexterity. The SIS assesses the health status and quality of life after stroke and consists of 59 items investigating 8 domains (strength, hand function, mobility, ADL, emotion, memory, communication, and social participation) and 1 question about the patient’s perception of their stroke recovery. The SIS physical domain is defined as the mean of final scores of four domains: Strength, hand function, mobility, and ADL. The SIS total score is defined as the mean of final scores of all the domains (including the SIS stroke recovery question). The MAL is a structured interview that investigates the upper limb amount of use (AOU) in everyday activities and their quality of movement (QOM) of 14 ADL items.

### 2.6. Data Analysis

Data from the exergame system and the assessments done by assessors were stored securely in a database at one of the rehabilitation sites. For the feasibility measures, descriptive statistics for the number of sessions, session duration, and exergaming duration, and session efficiency are reported. The session efficiency was calculated as the percentage of the actual amount of time spent using the exergames alone in a session over the total time of that session. For the safety measures, any occurrences of pain, fatigue, dizziness, and fall were reported. The number of dropouts was also documented. For the statistical analysis of the efficacy outcome measures, the IBM SPSS Statistics software package version 22 was used. We used the linear mixed model (LMM) analysis technique to model each outcome measure. In all the models, the fixed effects of the model were built using group, session, and their interaction. The baseline measurements (T0) were added to the models as covariates. The first-order autoregressive covariance structure with homogenous variances, i.e., AR(1), was found to be the most optimal choice for the models. For the post-hoc analysis, all the pairwise comparisons (simple effects) were adjusted using Bonferroni correction. For all inferential analyses, the probability of type 1 error was fixed at α = 0.05.

## 3. Results

Twenty-three stroke patients consented to participate in this study. Five participants did not complete the study. Among them, four participants withdrew from the study following consent but prior to the baseline evaluation, three from the intervention group and one from the control group. The reasons expressed were lack of time, general fatigue, and loss of motivation. In addition, one subject in the intervention group had restricted shoulder activities due to capsulitis and could not participate in the post-intervention and follow-up evaluations. Data regarding feasibility was reported but this patient was excluded from the efficacy analyses. Therefore, in total, 18 stroke subjects completed the full study (*n* = 9 per group). Table 1 shows the characteristics of the stroke subjects who participated in and completed this study.

Table 2 summarizes the results of the feasibility analysis. During the 4 weeks, subjects attended at least eight sessions of therapy using the exergames system with an average total duration of 44 min. From this 44 min of total duration, on average, half of it (21 min) was the actual time spent using the exergames. The other half was the time used for preparation, for displaying information on the screen to the subjects, for displaying results, for adjusting the gaming parameters, for resting if needed, etc. The session efficiency was variable among the subjects, ranging from 31% to 63% (standard deviation of 11%). During the gaming sessions, no serious adverse events were experienced by the intervention group. In all subjects, the maximum average perceived pain level on the VAS was below 2.1/10 and the maximum average perceived fatigue level on the Borg scale was below 3.5/10 during gaming sessions. The average perceived pain and fatigue for all subjects were 0.6/10 and 1.7/10, respectively (Table 2). There was no incidence of falls in any of the sessions and dizziness was reported only in one session for one subject.

Table 3 summarizes the statistical analysis performed on the efficacy data. In the FMA-UE and BBT, no significant differences between the two groups were found. In the AOU section of the MAL, no significant differences between the two groups were found. However, in the QOM section of the MAL, there was a significant effect of group (F(1,14) = 6.0, *p* = 0.029) and significant interaction between group and session (F(1,15) = 8.0, *p* = 0.013). The pairwise comparisons revealed that at T1, the intervention group had higher gains in the MAL-QOM score compared to the control group (mean difference = 1.0, F(1,20.8) = 11.9, *p* = 0.002) while the difference in gain (mean = 0.3) at T2 was not significant (F(1,20.8) = 0.8, *p* = 0.38). Looking at the intervention group alone, the gain in the MAL-QOM score from baseline was significant (*p* = 0.009).

In the mobility domain of the SIS, there was a significant difference between the two groups (F(1,15) = 7.2, *p* = 0.017). The mean difference between the intervention and control group was 5.5%. The same pattern was observed in the SIS-Physical where a significant difference between the two groups was present (F(1,15) = 5.0, *p* = 0.041). The mean difference between the intervention and control group was 6.7%.

**Figure 2:**
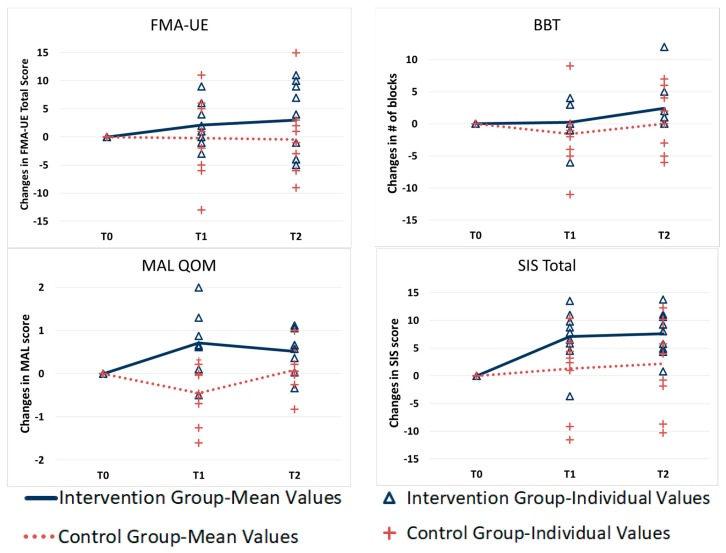
Average and individual change scores of the efficacy outcome measures at pre, post, and follow-up evaluations.

For the SIS-Total score, a trend in difference between the two groups was observed (F(1,15) = 3.9, *p* = 0.068) and the pairwise comparison showed a trend for greater improvement in the intervention group compared to the control group at both T1 (mean difference = 5.7, F(1,20.2) = 3.5, *p* = 0.077) and T2 (mean difference = 5.3, F(1,20.2) = 3.1, *p* = 0.094), although none of them were statistically significant. However, by looking at the intervention group, the changes of the SIS-Total from baseline to post-treatment were significant (mean difference = 7.1, *p* = 0.002) and were still present at the follow-up with a mean difference of 7.6 from baseline (*p* = 0.017). Figure 2 shows the changes in the FMA-UE, BBT, and QOM section of the MAL measure and SIS-Total score in both groups over time. As can be seen in Figure 2, the changes in the efficacy measures over time indicate that the intervention group had greater gains, although not all of them were statistically significant.

## 4. Discussion

In this pilot study, we examined the feasibility and safety of an exergaming system in a group of stroke survivors who were receiving usual out-patient rehabilitation services and explored its efficacy by comparing them to a usual out-patient care group. Meta-analyses examining the effect of the intensity of stroke rehabilitation on recovery suggest that the extent of recovery during the subacute stage post-stroke is related to the intensity of rehabilitation, i.e., the more time is spent in rehabilitation, the greater the extent of recovery [25]. However, following stroke, both therapists and patients primarily focus on regaining walking ability rather than restoration of upper limb function [26], resulting in less than 10 min of UE training in a rehabilitation session [27]. Therefore, any extra active UE training would be beneficial and in accordance to the best stroke practice guideline [2]. In our study, all participants in the intervention group attended exergaming sessions at least twice a week for 4 weeks, in addition to the rehabilitation services they were receiving. During that time, they were able to spend on average an additional 21 min of upper limb exercising in each 45 min session of exergaming with minimal therapist supervision. This high adherence rate to a rehabilitation intervention that supplemented ongoing rehabilitation suggests that stroke survivors in the sub-acute stage were able to participate in more rehabilitation than what rehabilitation centers are currently able to offer given financial and resource constraints.

In addition, this study showed that there were no adverse events when using the exergame system. The level of fatigue was low across sessions when using the exergame system. Furthermore, contrary to cyber sickness effects observed in immersive virtual environments [28], since this exergame system uses a non-immersive technology, we expected a very low rate of dizziness; indeed, only one instance of dizziness was reported during the course of study. No falls were reported at any time, although it is important to note that this exercise program was done while sitting. Therefore, findings from this study suggest that exergame systems can be used to supplement and hence increase the frequency of interventions for UE rehabilitation post-stroke, which is in line with recommendations from current stroke guidelines [2]. Increasing treatment intensity using exergames can be achieved in different ways. For example, in one of our local rehabilitation centers there is an exergame room that has been put into place, which provides additional therapy time for patients [29]. Such exergame rooms are likely cost and resource effective and could be part of the rehabilitation services provided.

Another objective of this study was to evaluate the efficacy of the exergame system for stroke rehabilitation. Even though this study was not designed nor adequately powered to detect between group differences, the intervention group showed a greater level of improvement, which in some cases was statistically significant, when compared to the control group. Compared to the usual out-patient care with no additional treatment, the supplement of the virtual reality exergame system showed an effect on self-reported health status and quality of UE use. In addition, by looking at the follow-up values as a measure of persistency, in none of the outcome measures did the average of the intervention group fall below average baseline values while this was the case in the control group. Future studies with larger sample sizes should examine the added value of such adjunct therapy.

While this study did not specifically examine the acceptability of the technology, findings regarding patient adherence to the intervention suggest that it was acceptable to patients to integrate exergaming during the rehabilitation phase, although further studies examining the factors impacting on patient and clinician acceptability, motivation, and adherence are needed.

In the current study, the exergaming system was used in clinical settings with therapists being present during the session even though the therapist’s role was only limited to adjusting the settings of the system and monitoring the patient’s progress. Used in this way, this could limit the acceptability for health care systems as no manpower is saved. However, this exergaming system is designed to be used as a tele-rehabilitation tool in home settings by individuals affected by stroke. The role of the therapist would then be to monitor patients’ progress and adjust the exergaming levels remotely; this would considerably reduce the therapist’s workload and may also contribute to acceptability by the patients. Therefore, use of this system as a tele-rehabilitation tool should be investigated in future studies.

### Study Limitations and Future Directions

In this clinical research study of the rehabilitation exergaming system, the sample recruited was limited to patients without additional deficits that commonly occur following a stroke, such as hemianopsia, hemineglect, and cognitive deficits, when these could affect the ability to play the game. Therefore, findings from this study cannot be generalized to all stroke survivors. Future larger studies should include and stratify patients with different clinical characteristics. Given the nature of the intervention, patients could not be blinded, hence a single-blind design was proposed. While the small sample size of this study would not provide sufficiently robust data regarding the efficacy of the exergame system, it has provided information regarding its feasibility and safety and is the basis for future larger-scale clinical trials for the sample size requirements and the possible variability of the data. In addition, qualitative data regarding patients’ perception of and satisfaction with the exergaming system, while important to help better understand findings related to the use of such a program, were not measured in this study and is a limitation of this study. Future studies should include such subjective data.

In this study, there was a trend for maintaining gains by some participants in the follow-up period, but this was not consistent across the outcome measures. Further studies are needed in order to understand patient characteristics that impact on how gains are maintained over time. Furthermore, studies with long-term follow-ups are needed to determine the extent to which gains in motor control, activities, and participation are maintained and whether continued training would be beneficial to maintain or increase gains. Further studies are also needed to identify the ideal treatment intensity and duration.

## 5. Conclusions

This study showed that use of the exergaming system with minimal therapist supervision as a supplement to out-patient rehabilitation interventions for stroke patients in a sub-acute and chronic stage is safe and feasible, and may improve upper limb use.

## Figures and Tables

**Table 1:** Characteristics of the stroke participants.

	Intervention Group (*n* = 9)	Control Group (*n* = 9)
**Sex (Male:Female)**	5:4	5:4
Age in Years (Range) *	42.2 ± 9.5 (22.7–52.6)	57.6 ± 10.5 (41.7–73.6)
**Months Poststroke (Range)**	5.7 ± 3.2 (2.6–12.9)	8.4 ± 7.8 (2.0–23.3)
**Handedness (Right:Left:Ambidextrous)**	9:0:0	8:0:1
**Side of Hemiparesis (Right:Left)**	4:5	3:6
**Stroke Type (Ischemic:Hemorrhagic)**	7:2	8:1
**Baseline Measurements (T0)**		
**Chedoke McMaster Upper Extremity**	4.2 ± 1.3 (3–6)	4.0 ± 1.1 (3–6)
**Fugl-Meyer Assessment Upper Extremity (FMA-UE)**		
** A. Upper Extremity**	25.6 ± 9.1 (12–36)	26.9 ± 5.6 (21–36)
** B. Wrist**	5.3 ± 4.3 (0–10)	6.8 ± 3.2 (0–10)
** C. Hand**	9.4 ± 5.6 (1–14)	10.3 ± 3.4 (3–14)
** D. Coordination/Speed**	3.9 ± 1.3 (2–6)	4.0 ± 1.7 (2–6)
** Total**	44.2 ± 18.8 (15–63)	48.0 ± 11.4 (28–62)
**Box & Block Test of Paretic Hand (number of blocks per minute)**	27.0 ± 23.5 (0–54)	33.1 ± 14.3 (2–48)
**Upper Extremity Motor Activity Log—Amount of Use (/5)**	1.9 ± 1.4 (0.0–4.1)	2.0 ± 1.3 (0.5–4.9)
**Upper Extremity Motor Activity Log—Quality of Movement (/5)**	2.9 ± 0.7 (2.0–4.0)	3.4 ± 0.8 (2.3–4.9)
**Stroke Impact Scale—Physical Domain (/100)**	63.3 ± 17.2 (34.0–82.7)	68.7 ± 14.4 (44.1–87.1)
**Stroke Impact Scale—Total (/100)**	68.2 ± 14.5 (40.4–82.7)	71.3 ± 10.8 (54.6–88.7)

The numbers beside ‘±’ sign represent mean ± standard deviation. The numbers in parentheses represent range. (/x) indicates maximum of the scale. * Difference in the baseline between the two groups is significant (*p* < 0.05).

**Table 2:** Summary of the feasibility and safety outcome measures.

ID	# of Sessions	Total Session Duration (min)		Total Exergaming Duration (min)		Session Efficiency (%)		Pain Level (VAS)		Fatigue (Borg Scale)		# of Falls	# of Dizziness
		Mean	Std	Mean	Std	Mean	Std	Mean	Std	Mean	Std		
**1**	8	50	6.0	26	5.3	53%	12%	0.0	0.0	0.6	0.9	0	0
**2**	8	47	3.7	23	3.7	50%	10%	0.4	0.7	3.4	0.7	0	0
**3**	8	41	3.2	16	2.9	40%	8%	0.0	0.0	1.6	1.4	0	0
**4**	9	42	2.6	13	3.0	31%	7%	0.8	1.7	0.3	1.0	0	0
**5**	11	42	5.1	22	4.1	54%	9%	0.0	0.0	2.9	1.7	0	0
**6**	8	44	3.2	18	2.8	41%	6%	0.4	0.5	1.2	0.5	0	0
**7**	8	42	4.2	24	4.9	56%	7%	1.4	0.9	1.3	0.7	0	1
**8**	9	48	5.8	29	5.0	62%	5%	0.0	0.0	1.8	1.0	0	0
**9**	8	43	4.5	26	3.1	63%	4%	1.1	1.4	1.5	0.5	0	0
10 *	8	44	3.2	17	3.5	39%	5%	2.1	1.0	2.0	0.0	0	0
**All subjects**		**44**	**3.1**	**21**	**5.3**	**49%**	**11%**	**0.6**	**0.7**	**1.7**	**0.9**		

* This participant is excluded from the subsequent analyses as the follow-up evaluations were not completed.

**Table 3:** Values of the efficacy outcome measures over time.

Outcome Measures	Baseline (T0) ^†^	Post-Intervention (T1) ^‡^	4-Week Follow-Up (T2) ^‡^	Change from T0 to T1 ^@^	Change from T0 to T2 ^@^
**Intervention Group (*n* = 9)**					
**FMA-UE: Total**	44.2 ± 18.8	46.3 ± 16.8	47.2 ± 14.7	2.1	3.0
**MAL-AOU**	1.9 ± 1.4	2.1 ± 1.7	2.3 ± 1.7	0.2	0.4
**MAL-QOM**	2.9 ± 0.7	3.6 ± 0.7 *	3.4 ± 0.8	0.7 *	0.5
**BBT**	27.0 ± 23.5	27.2 ± 22.8	29.4 ± 24.6	0.2	2.4
**SIS Strength**	52.1 ± 15.0	56.3 ± 16.8	57.6 ± 18.4	4.2	5.6
**SIS Mobility**	85.2 ± 14.7	91.0 ± 10.4 *	91.7 ± 10.3 *	5.9	6.5
**SIS Hand Function**	41.7 ± 33.6	52.2 ± 38.6	51.1 ± 34.9	10.6	9.4
**SIS ADL**	74.4 ± 16.4	80.0 ± 14.8	78.4 ± 12.7	5.6	4.8
**SIS Physical**	63.3 ± 17.2	69.9 ± 18.3 *	69.2 ± 17.1 *	6.5 *	5.9
**SIS Memory**	93.3 ± 6.8	97.2 ± 3.9	97.6 ± 5.1	4.0	4.4
**SIS Emotion**	67.0 ± 15.7	75.3 ± 22.2	81.5 ± 15.2	8.3	14.5
**SIS Communication**	90.1 ± 14.3	92.5 ± 14.1	94.0 ± 9.4	2.4	4.0
**SIS Social Participation**	54.2 ± 27.3	67.4 ± 24.8	61.1 ± 23.4	13.2	6.9
**SIS Stroke Recovery**	55.6 ± 23.1	65.6 ± 20.1	70.0 ± 22.1	10.0	14.4
**SIS Total**	68.2 ± 14.5	75.3 ± 14.9	75.8 ± 14.0	7.1 *	7.6 *
**Control Group (*n* = 9)**					
**FMA-UE: Total**	48.0 ± 11.4	47.8 ± 12.3	47.6 ± 13.3	−0.2	−0.4
**MAL-AOU**	2.0 ± 1.3	2.1 ± 1.0 *	1.9 ± 0.9	0.0	−0.1
**MAL-QOM**	3.4 ± 0.8	2.9 ± 0.7 *	3.5 ± 0.8	−0.5	0.1
**BBT**	33.1 ± 14.3	31.6 ± 14.7	33.1 ± 12.5	−1.6	0.0
**SIS Strength**	59.7 ± 19.8	51.4 ± 14.9	59.0 ± 22.1	−8.3	−0.7
**SIS Mobility**	82.7 ± 14.2	85.2 ± 10.1 *	83.3 ± 14.0 *	2.5	0.6
**SIS Hand Function**	53.9 ± 28.6	55.0 ± 25.1	61.1 ± 32.8	1.1	7.2
**SIS ADL**	78.6 ± 16.0	75.6 ± 14.3	73.9 ± 19.9	−3.1	−4.7
**SIS Physical**	68.7 ± 14.4	66.8 ± 13.4 *	69.3 ± 18.3 *	−2.0	0.6
**SIS Memory**	83.7 ± 14.4	82.1 ± 21.1	86.1 ± 19.4	−1.6	2.4
**SIS Emotion**	69.1 ± 13.2	80.2 ± 9.9	78.7 ± 14.2	11.1	9.6
**SIS Communication**	87.7 ± 13.7	88.9 ± 12.6	83.3 ± 21.2	1.2	−4.4
**SIS Social Participation**	60.1 ± 17.1	64.6 ± 23.9	63.9 ± 17.9	4.5	3.8
**SIS Stroke Recovery**	66.1 ± 15.4	70.6 ± 14.9	71.7 ± 18.2	4.4	5.6
**SIS Total**	71.3 ± 10.8	72.6 ± 11.1	73.5 ± 14.7	1.3	2.2

Abbreviations: FMA-UE, Fugl-Meyer Assessment-Upper Extremity; MAL, Motor Activity Log; AOU, Amount of Use; QOM, Quality of Movement; BBT, Box and Block Test; SIS, Stroke Impact Scale. ^†^ *p*-values noted in this column are for simple comparisons between the intervention and control groups. ^‡^ *p*-values noted in these columns are for between-group comparisons using Linear Mixed Model approach and setting baseline as a covariate. ^@^ *p*-values noted in these columns are for testing changes between baseline and the specified time point in each group. * *p* < 0.05. The numbers beside ‘±’ represent mean ± standard deviation.
